# The Sixth Annual Meeting of the Dental Society of the State of N. Y.

**Published:** 1874-08

**Authors:** 


### THE SIXTH ANNUAL MEETING OF THE DENTAL SOCIETY OF THE STATE OF NEW YORK.

This Society met in the Assembly Chamber of the Capitol at Albany, on the twenty-fourth of June, and held a most pleasant and profitable meeting.

Dr. J. G. Ambles, of New York presided, and papers were read as follows:

Dental Education, by W. H. Atkinson.

The Histology and Nutrition of Deciduous Teeth, by W. C. Barrett.

Preserving the Teeth, by O. A. Jarvis.

Chemical and Galvanic action upon Teeth, by S. B. Palmer.

The Board of State Censors reported, that the applicants for the diploma of the Society were less in number than usual.

They present the name of but one, Ira C. Curtiss, Fulton, Oswego Co., as having passed the requisite examination.

A number of cases of the unwarranted use of the title of M. D. S. (Master of Dental Surgery) were reported by the Committee on Law, and it was ordered by the Society that they be prosecuted for infringement of the Law.

The Board of Censors made a supplementary report recommending that the Society appoint a committee to wait upon the Legislature and ask to have the dental law so amended as to make it equally stringent with the late medical law, but upon consideration the subject was tabled for a year.

The following are the officers elect for the ensuing year:

President, W. C. Barrett, Warsaw;

Vice-President, L. S. Straw, Newburgh;

Rec. Secretary, Charles Barnes, Syracuse;

Cor. Secretary, W. S. Elliott, Goshen;

Censor, L. H. McCall, Binghampton, 6th Dist.;

Censor, L. F. Harvey, Buffalo, 8th Dist.;

Permanent Members:

Chas. Merritt, 1st Dist.; W. F. Shannon, 2d Dist.; A. S. Roberts, 5th Dist.; B. R. McGregor, 7th Dist.; W. C. Barrett, 8th Dist.

